# Assessment of a Person’s Emotional State Based on His or Her Posture Parameters

**DOI:** 10.3390/s23125591

**Published:** 2023-06-15

**Authors:** Yulia Shichkina, Olga Bureneva, Evgenii Salaurov, Ekaterina Syrtsova

**Affiliations:** Department of Computer Science and Engineering, Saint Petersburg Electrotechnical University “LETI”, 197022 Saint Petersburg, Russia

**Keywords:** human emotion, human–machine interaction, automatic emotion recognition, psychological diagnosis, posture recognition, body pressure distribution, pressure sensing, co-evolutionary hybrid intelligence

## Abstract

This article is devoted to the study of the correlation between the emotional state of a person and the posture of his or her body in the sitting position. In order to carry out the study, we developed the first version of the hardware-software system based on a posturometric armchair, allowing the characteristics of the posture of a sitting person to be evaluated using strain gauges. Using this system, we revealed the correlation between sensor readings and human emotional states. We showed that certain readings of a sensor group are formed for a certain emotional state of a person. We also found that the groups of triggered sensors, their composition, their number, and their location are related to the states of a particular person, which led to the need to build personalized digital pose models for each person. The intellectual component of our hardware–software complex is based on the concept of co-evolutionary hybrid intelligence. The system can be used during medical diagnostic procedures and rehabilitation processes, as well as in controlling people whose professional activity is connected with increased psycho-emotional load and can cause cognitive disorders, fatigue, and professional burnout and can lead to the development of diseases.

## 1. Introduction

Emotions reflect a person’s relation to various situations or events. They implement feedback from a person to the objects of the external world; emotion is an important component of the social environment and a marker of human interaction with various information systems [1]. Recognition of emotions is actual in education, medicine, social sciences, and entertainment and has a special importance for human interaction with complex technical objects where the human factor is significant and determines the safety of operation. Therefore, the development of devices and systems that can recognize, process, interpret, and simulate human emotions is essential. This problem is solved within the framework of affective computing [2], where various models of emotions and effective mathematical methods for their extraction, classification, and analysis have been created.

According to Scherer’s theory [3], emotions consist of five related components: cognitive (appraisal), neurophysiological (bodily symptoms), motivational (action tendencies), motor expression component (facial and vocal expression), and subjective feeling component (emotional experience). Thus, emotions can be identified and assessed by analyzing these components. Currently, all of the above methods for assessing emotional states, both those based on subjective assessments by specialists in psychology and those associated with the development and application of hardware and software systems, are being actively developed.

Hardware methods for obtaining information about a person’s emotions can be classified into two groups [4]. The methods of the first group are based on physiological parameters [5] obtained on the basis of electroencephalogram (EEG) [6,7,8], electrocardiogram (ECG) [9,10], and electromyogram (EMG) [11,12]. Emotions can also be assessed based on temperature [13,14], skin-galvanic reaction, breathing patterns, heart rate [15,16,17], etc. In Ref. [18], a connection between emotions and involuntary human body movements (tremor) was proved. This makes it possible to assess emotional states based on tremor parameters [19]. Methods based on physiological signals allow obtaining data about emotional changes in real time with a high level of accuracy. However, these methods often require overlaying sensors on the human body, in some cases requiring expensive and large-sized devices. This limits the use of these methods, for example, in systems controlling the interaction of a person with technical objects. The second group of methods is based on behavioral reactions assessed by facial features, such as mouth activity, head movements, blink frequency, spatial distribution of gaze, pupil dilation, and eye movements [20,21,22,23]; voice [24,25,26]; and movements, gait, and body postures [27,28,29]. Currently, research on emotion recognition has mainly focused on facial expression and physiological cues, while emotion recognition based on the modality of posture has been investigated little.

The aim of our research was to find a correlation between the emotional state and the posture of a sitting person. To perform the research, we designed a special posturometric chair, which allows us to monitor the parameters of the sitting person’s posture. We also developed special software to provide a mark-up in time of continuously measured pose parameters in accordance with changes in the emotional state, controlled by a professional psychologist.

In our work, we focused on the study of the relationship between the emotional state of a person and his or her posture in the sitting position. Our main contributions are as follows:

- We developed a posturometric chair to detect sitting posture (body tilt, weight distribution, degree of leaning on the backrest) using sensors embedded in the seat and backrest.
- We applied and compared the effectiveness of several machine learning methods in the task of emotion recognition from sitting posture.
- We have proposed an approach to estimating the emotional state of a person based on the body position changes of the person sitting in the armchair, based on the concept of co-evolving hybrid intelligence, which will allow taking into account the individual characteristics of the tested people.
- We formed the basis of a database of sitting postures characterizing six emotions classified according to three levels of manifestation.

The importance of our research is due to the use of the detected dependencies; it is possible to estimate the emotional state of a seated person without the application of sensors to his body. This allows us to analyze the emotional states of, for example, fatigue or drowsiness of a driver or operator of complex equipment, and to generate warnings for that person. The results obtained can also be used for mental health rehabilitation. The created system can be a part of other more complex systems that implement human–machine interaction and especially those where personalization is important.

The novelty of our study is determined by the fact that we used an original design and simple sensors to create a posturometric chair, trained known machine learning models, and described a personalized approach, which together allowed us to efficiently solve the problem of emotion classification.

The work is structured as follows. First, in Section 2 we briefly review typical related works presented in the literature. In Section 3 we describe the developed equipment for assessing the characteristics of human posture during various emotional states and show the method of its application. Section 4 shows the results of the usage of our hardware–software tools during the experiment and the analysis of the obtained results. Section 5 provides a comparison with existing studies, shows the limitations of the proposed approach, and identifies areas for further work. The conclusion is given in Section 6.

## 2. Literature Review

One of the first studies of emotion recognition by posture is presented in [30]. M. Coulson developed 176 computer-images mannequins for the postural expressions of six emotions. In the models describing a standing person, the center of mass displacement and six joint rotations (head bend, chest bend, abdomen twist, shoulder adduct/abduct, shoulder swing, and elbow bend) were taken into account. The posture was assessed from three different viewpoints. The results of the pose analysis by this method and the methods of speech analysis and facial expressions for human emotion evaluation correlate with insignificant discrepancies. For the models created, it was determined how different emotions are assigned to body postures, the influence of human anatomical features and the position of observation were taken into account. The nature of confusion in emotion recognition was determined.

There are a number of methods based on the peculiarities of human posture by means of Laban movement analysis (LMA), which divides human body posture into four components: body, effort, form, and space [30]. Laban’s method was developed to analyze dance technique and described a direct connection between a person’s internal state (experiences, emotions) and posture or movement. This method was used as the basis for the development of automatic pose recognition and emotion analysis systems. A. Aristidou et al. [31] coded human posture based on LMA and proposed a set of 86-dimensional posture features. Speed, acceleration, and distance to various key points of the human body were used to describe the pose. The classification accuracy using machine learning methods such as random forest, extremely randomized trees, and support vector method exceeded 90%. X. Fu et al. [32] redefined key points of the human motion model extended dynamic functions based on pose encoding and created an 80-dimensional list of functions that can fully describe the human pose. After conducting experiments with different neural network models, the authors obtained a recognition accuracy of 72.16% in the case of classification into four classes. The authors of [33] showed that static human body posture can be described by a body factor and a shape factor based on the LMA method. The authors chose to encode the human body pose using 38-dimensional functions, and this simplified the description. An improved variant of VGG16 (VGG16 is a variant of the VGG model with 16 convolution layers) was proposed by S. Wang et al. [34]. The above methods are based on video analysis, and their implementation requires significant computing resources and time.

Methods of human posture recognition based on sensors are easy to implement. Various kinds of optical sensors, ambient light sensor, and curvature sensor, etc., can be used for this purpose [35]. Good posture assessment results can be obtained using pressure sensors. The authors of [36] investigated foot movements and posture characteristics in the sitting position to detect acute stress. Pressure-sensitive insoles were used to sense the distribution of pressure on the foot. The pressure was monitored using 16 sensors placed at critical pressure points. In this system, the level of stress calculated using machine learning models correlated with the level of stress reported by the subjects with a coefficient of 0.79. K. Bourahmoune et al. [37] proposed a solution for intelligent posture training based on accurate real-time monitoring of seated posture using a LifeChair IoT cushion and supervised machine learning based on pressure measurement and user body data. Their system’s performance in seated posture recognition tasks is over 98.82% in recognizing 15 different seated postures.

A good quality of classification of a sitting pose can be achieved by embedding the pressure sensors directly into the chair. In Ref. [38], a special textile sensor with 240 sensing elements was proposed for classification of sitting postures on a chair. The implementation of this idea is limited by the complexity of manufacturing the sensor. A chair with built-in sensors was developed by the authors [39] for real-time monitoring and classification of the seated worker’s posture. The chair system is a mixed sensor system using six pressure sensors and six infrared reflective distance sensors. Using the k-nearest neighbor algorithm, the mixed sensory system classified posture as one of the posture categories defined on the basis of sitting ergonomics or sitting-related musculoskeletal problems. Emotion assessment is not performed in this system. Assessment of the stress level using the chair is shown in article [40]; in the article the authors showed that the characteristics of the pressure distribution on the chair are related to the affective states. Important for our study is the fact that B. Arnrich and co-authors established that a person-independent discrimination of stress from cognitive load is feasible when using pressure data only. Q. Hu and co-authors in article [41] report on a system of posture recognition on an office chair that can classify seven different sitting postures related to musculoskeletal health. The system uses six flexible sensors, and the processing is based on the application of a machine learning algorithm of a two-layer artificial neural network implemented on a field-programmable gate array.

The system we propose has certain advantages in comparison to the works described above. Our system is based on the use of simple sensors; it does not require placing sensors on the human body. The volume of information generated by HW is much smaller than in the video analytics systems, which simplifies the data processing. Machine learning models are based on data that cover the user’s back and lower torso areas and allow for the assessment of emotional state.

## 3. Materials and Methods

To carry out research, a posturometric armchair with pressure-sensitive sensors was made, and a number of studies to assess the relationship between sensor readings and the emotional state of a person were performed; a system for identification of emotions and their physical manifestations in humans, based on the use of artificial intelligence technologies, was prepared and initially tested.

### 3.1. The Concept of Co-Evolutionary Hybrid Intelligence and a Cognitive Architecture for Its Implementation

The analysis of the interaction between AI and the environment is presented in many works. One of the first studies devoted to the interaction between AI and machine is presented in [42]. The author emphasizes that humans and AI should be considered as a system. The author argues that the effectiveness of the system can be improved by considering the whole as a set of interacting components, rather than considering the components individually. He also proves that the system should provide the ability to adapt augmentation tools to individual human characteristics.

In the concept of co-evolutionary hybrid intelligence (CHI) [43,44] that we are developing, we emphasize that humans and AI must be considered as a single system, where the following factors are taken into account:

- Participants and elements of the system should have cognitive interoperability;
- The system has to be self-developing;
- The system and its participants must have the property of self-reflexivity;
- The feedback in the system should be obligatory;
- The participants of the system should contribute to the development of each other and the system as a whole.

To implement CHI, we propose the cognitive architecture described in Ref. [44]. The main difference between the cognitive architecture for CHI and existing ones (such as [45]) is the inclusion of humans in the system and ensuring the co-development of humans and AI. Humans are viewed as both a subject and an object at the same time. As a subject, the person acts and affects the operation of the system. As an object, the person is a component of the system which has its own dynamic characteristics, changing in the process of system operation. The main components of the cognitive architecture for CHI are as follows:

- Data sources. These are primary sources representing data about a person and various objects of his environment. In our system of an assessment of a person’s emotional state, such sources are sensors of a posturometric armchair, a video camera, and sensors for monitoring of the temperature and parameters of breathing of the person. Sources also can be various databases and other means of storing and accumulating information. In this article we show processing of data from only one source—a group of sensors of a posturometric chair.
- Narrow AI. A module that implements preprocessing and data processing methods. In our case, it provides primary filtering of sensor signals, which we describe in the article, and video preprocessing.
- Multimodal data. Multimodal data processing is implemented in a module that collects data about the object at a given point in time from different sources, coordinates these data on a timeline or other scale, and selects from the whole set of data the ones that are relevant to the current situation.
- Hybrid system identification. This operation is realized in the module of state parameters identification of the system and its individual components. For example, in our system, this module can identify parameters of the human state.
- Activity models. The activity model module processes the existing models of actions in the system. For example, in our system, this module selects appropriate decision-making models depending on the known parameters and generates action scenarios.
- Generalized modeling module. The generalized modeling module handles the models, refines them, and combines them into a generalized system model.
- Generative AI. The module provides modification of existing models and creation of the new ones.
- Decision-making and action planning. In this module, an optimal model is selected from a set of models, and a single decision is chosen from a group of decisions. Decisions can have different characters. For example, if some condition is defined, it is a fixation of the fact, and if the scheme of correction of a condition is offered, it is some plan. Other variants of decisions are also possible.
- Execution and management. The module provides the execution of an action plan, if there is a need. In our system, it can be, for example, automatic adjustment of the chair to the position of the person, taking into account his or her current state and the target state. This module is currently under development.

In this article, we present only part of the modules of the system based on the described cognitive architecture described above. These modules are a very important part, ensuring the development of CHI. The main CHI feature is not the adjustment of artificial intelligence to humans, but their joint interaction and cognitive activity. Based on the obtained data about the human condition, the intelligent system must form responses (e.g., explanations, recommendations, alerts) in such a way that the human adequately perceives the system’s actions and uses the interaction experience with AI for its own development. There is also a reverse impact on the human intellectual system; it is realized in the form of new knowledge, interpretations, explanations, and other information that can be clarified through dialogue with the system. The quality of this dialogue also depends on the psychophysiological state of the person. Both existing theories of knowledge representation, such as product modeling or semantic networks, and a new apparatus based, for example, on the theory of multi-operations can be used to organize the feedback. Therefore, the research is important not only for the application domain, but also for the development of intelligent systems in general.

### 3.2. System for Defining the Characteristics of the Human Body Position

**Figure 1:**
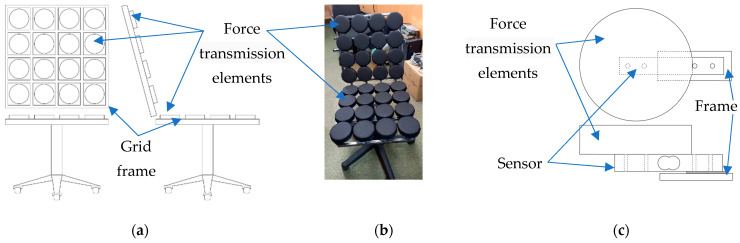
Posturometric armchair for assessing a person’s functional state: (**a**) diagram of transmission element’s location in the backrest and seat; (**b**) overall view of the armchair; and (**c**) sensor-mounting design on the grid frame.

Figure 1 shows the designed posturometric armchair, allowing us to obtain information about the posture of the sitting person. The back and seat of the chair are grids with strain gauges installed in the cells; 16 strain gauges are installed in the seat and 16 sensors are placed in the backrest (Figure 1a,b).

The straight bar load cell has been selected for the measurement. The straight rod load cell is made of aluminum alloy and can detect impacts up to 10 kg. The load cell has four mounting holes on one plane and a special twin hole on the other plane. In the area of the double hole, there are strain resistors, sealed with silicone mastic. When the sensor is mounted, one side of the sensor is rigidly fixed to the grid frame and the other side is fixed to the force transmission element, as shown in Figure 1c. A seated person acts on the force transmission element; this causes sensor bending and a corresponding change in resistance of the strain resistors connected in a bridge circuit.

**Figure 2:**
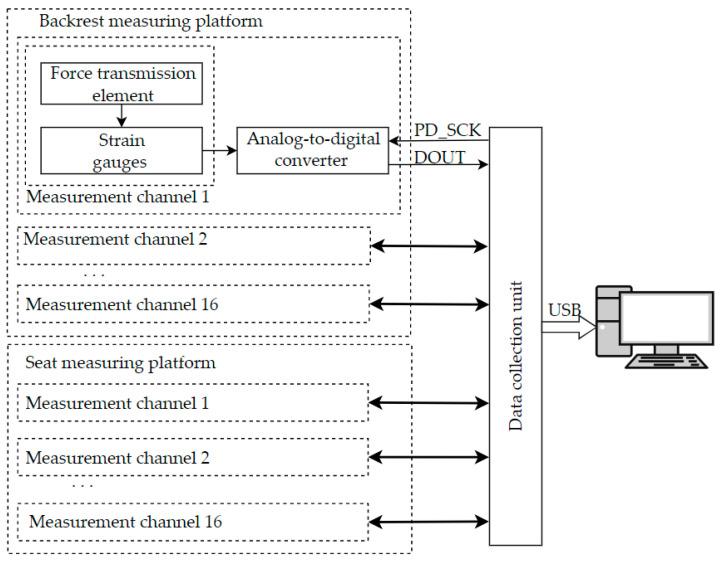
Architecture of the measuring system of the posturometric armchair.

Figure 2 shows the architecture of the measuring system. To collect information about the impact on the gauges in the system, there are 32 measuring channels operating in parallel. Each channel includes the following elements:

- A force transmission element transmitting the force exerted by a sitting person to the strain gauge.
- The strain gauge with built-in bridge generates an analog signal proportional to the applied force. The outputs of the supply and measurement diagonals of the bridge are connected to the digitizer unit through a cable shortened to minimize interference. In the measuring system, sensors with the following characteristics are used: maximum permissible weight—10 kg; error—0.05%; working temperature—−10–50 °C; supply voltage—3–12 V.
- The analog-to-digital converter provides the digitization of the bridge mismatch signal. In the system, HX711 converters are used. The converter has a built-in multiplexer that allows communication with one of the two input channels. Channel A can be programmed with a gain of 128 or 64; channel B has a fixed gain of 32. The chip has a built-in voltage regulator, eliminating the need for an external regulator. The clock input is multifunctional; in this design we use it to supply external clock pulses.

The result of digitizing the bridge mismatch signal is a binary code characterizing the mechanical action applied to the force transmission element. The resulting digital code is transmitted via a two-wire interface to the data collection unit realized on the Arduino processor. During the analog-to-digital conversion, the digital output DOUT of the ADC stores a logical «1», while the data acquisition unit ensures that the logical «0» is stored at the PD_SCK line. A «0» signal on the DOUT output indicates that the conversion is complete and the data are ready to be extracted. When the data are ready, 25 pulses are sent to the ADC input PD_SCK and the ADC responds with a serial 24-bit code corresponding to the result of the conversion through the DOUT line.

The data collection unit is realized on the Arduino MEGA board. At the beginning, the processor waits for the readiness signal from the first sensor; after detecting a low-level signal on the information output of the sensor, the processor performs a serial poll. Based on the data received from all sensors, the data collection unit generates the frames and transmits them to a personal computer via the USB interface.

**Figure 3:**
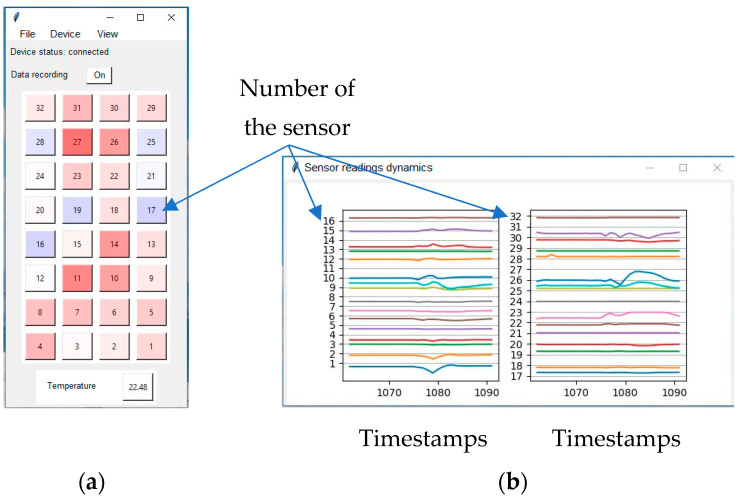
Working windows of the program: (**a**) visualization of the sensor readings; and (**b**) visualization of the sensor readings dynamics.

Special software is prepared for controlling the armchair operation from a PC; it provides setting of the measuring channels, reading of the results, primary processing, and visualization of the data. It is written in Python 3.9.13 using the Tkinter 8.6 graphic library. The working window of the program is shown on the Figure 3. There are two operating modes in the program:

- Calibration mode. In this mode, the sensors are polled without any influence on them to form corrective values, used to compensate for the additive error.
- Measuring mode. In this mode, the results of measurement are read out. To control this mode, the signals for starting the sensor polling and stopping the polling are used. In this mode, 10 measurements per second were performed, then averaged in accordance with the sensor manufacturer’s recommendation. Thus, the frequency of measurements was 1 sample per second.

In the program window shown in Figure 3, each cell shows the result of sensor actuation. When the sensor reading changes, resulting from pressure on the sensor, the cell smoothly changes color to red. When it returns to its original state, which corresponds to the removal of pressure from the sensor, the cell changes color to blue. In addition, there is a temperature sensor installed in the chair, and the data from it are displayed in a separate cell in digital form. For each sensor, you can additionally monitor the changes in readings in a separate window.

### 3.3. Formation and Identification of an Emotional State

There are various approaches to classifying emotions, according to which six [46], seven [47], or eight [48] emotions are distinguished, and their strength is assessed separately. These highlighted basic emotions are relevant to all ages and cultural differences. Currently, various methods are used to assess the emotional state that do not require special equipment:

- Questionnaire-based assessment, for example, using Spielberger anxiety tests and their modifications [49], the subjective MF 20 asthenia assessment scale [50], Holmes and Ray’s stress tolerance and social adaptation methodology [51], the Medical Outcomes Study-Short Form (MOS SF-36) health assessment questionnaire [52], and so on;
- Analysis of visual information, performed both by a specialist and automatically, for example, using neural network technologies.

In our study, the control of the subject’s emotional state and the estimation of this state were carried out visually by a specialist psychologist on the basis of a method built on a combination of the principles of modern psychodiagnostics [53] and the techniques of hypnosuggestive psychotherapy [54]. These methods make it possible to evoke and then identify six basic emotions that people experience from birth, regardless of cultural affiliation. These are the following emotions:

1. Joy—feeling happy, cheerful, enjoying, contentment, bliss, pride, excitement, fascination, pleasure, euphoria, acceptance, friendliness, trust, kindness, sympathy, enthusiasm, admiration.
2. Sadness—concern, joylessness, regret, guilt, shame, loneliness, sadness, despair.
3. Fear—anxiety, apprehension, nervousness, uneasiness, fright, misgivings, suspicion, doubt, suspense, terror, panic.
4. Anger—irritation, resentment, indignation, hostility, annoyance, nervousness, aggression.
5. Disgust—contempt, disdain, aversion, dislike.
6. Astonishment—amazement, excitement, shock.

According to the authors of [55], there are basic emotions, and all other emotions are mixed, in that “they can be synthesized by various combinations of the primary emotions”. Izard C. [56] also believes that there are basic emotions, and all other emotional states are derivative or composite, i.e., arise on the basis of several fundamental ones. For this reason, in our study it was decided to begin by training a system for recognizing basic emotions.

### 3.4. Testing Procedure

A number of 25 people aged 20 to 23 voluntarily participated in this study as subjects. All study participants were informed of the details of the experiments, including the main experimental tasks. Participants were told that the research aimed to investigate behavioral changes when performing tasks that elicited different emotional states. Informed consent to participate in the experiment was obtained from each participant. The research protocol was reviewed and approved by the Ethical Committee of the Human Brain Institute of the Russian Academy of Sciences 31 May 2022.

**Figure 4:**
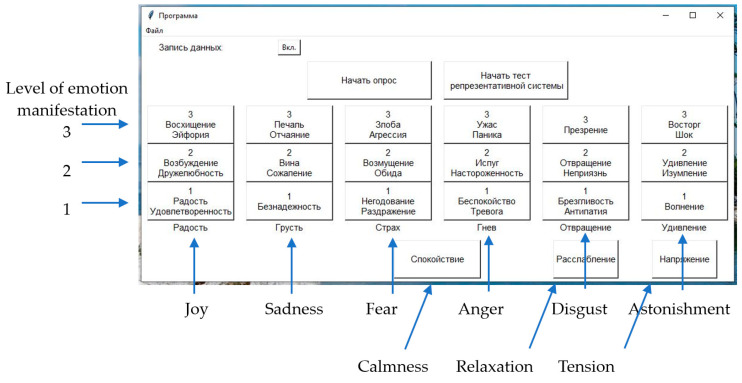
Panel of emotions—the main window of the experiment operator’s program for marking up the sensor readings when different emotions are evoked in a person.

The procedure of the experiment was as follows. The participant sat in a chair. The operator, using the emotional bridge technique of Eriksonian hypnosis [57], provoked various emotions in the subject and registered them. Special software was used to record the emotions observed in the subject. The working window of the operator’s program is shown in Figure 4. This window is a panel of emotions; the main elements of the window are the buttons corresponding to the six emotions, whose manifestations are evaluated on a three-level scale. Additionally, in the window there are buttons marking the states of calmness, relaxation, and tension, necessary for fixing the states of the person between active manifestations of the evoked emotions.

Provocation of certain emotional states involved the following stages:

1. Modeling of the initial neutral state in the subject and fixation of the state on the panel of emotions.
2. Reproduction of the necessary emotional state.
3. Return of the subject to the initial state of comfort.

These stages were repeated with the subject’s reproduction of the following states: joy, sadness, fear, anger, disgust, and astonishment.

A total of 25 people took part in the study. The following equipment was used in the experiment: camcorder, microphone, keyboard, mouse, electrocardiography sensor (ECG), galvanic skin reaction sensor (GSR), posturometric armchair, and software and hardware for synchronization of state parameters records of the subject.

## 4. Processing of Measurement Results

The data obtained on the basis of sensor signals were arranged in accordance with the observed emotions, taking into account their level on a scale from 1 to 3. During monitoring, the moments of change of the observed emotion were fixed, and the initial level (absence of emotion or the calm state of the person) was taken as 0. Between the marks showing change of the emotion, the emotional state of the person was considered as unchanged. The time of the experiment was three to three and a half hours. This allowed us to obtain a statistically significant amount of posturometric information for six emotions divided into three levels for each participant of the experiment.

**Figure 5:**
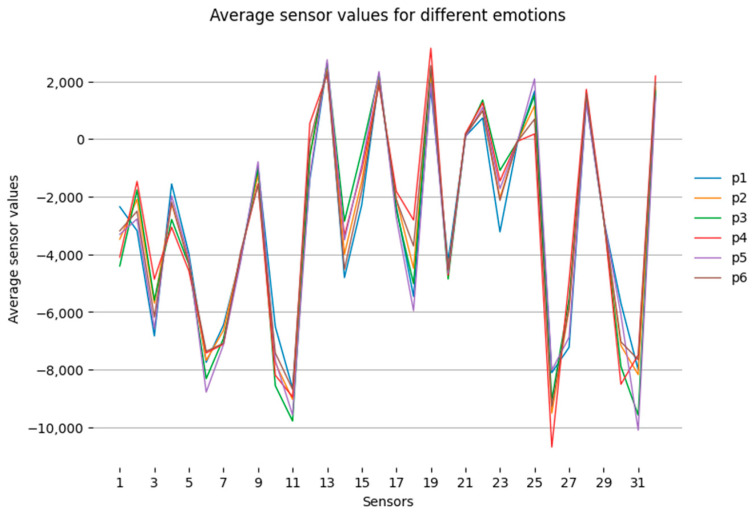
Average values of sensor indications by emotions for the group of subjects: p1—joy, p2—sadness, p3—fear, p4—anger, p5—disgust, p6—astonishment.

The primary analysis of the information was performed using statistical methods. The values of the sensor signals for a particular emotion were selected, and the mean and maximum values, as well as the standard deviation for each emotion, were calculated. The calculations were performed using the mean, max, and std methods built into the NumPy library. The mean value shows the general tendency of influence on a certain sensor and allows us to estimate its «typical» value under the tested emotion. The maximum value of the sensor signal at the tested emotion shows the upper limit of influence on a particular sensor. The standard deviation shows the degree of variability or uncertainty in the effect on the sensors. Figure 5 illustrates the averaged digitized sensor values by emotion for a group of subjects. Analysis of the figure shows that the average values for the emotions are correlated with each other. The correlation coefficient between them ranges from 0.72 to 0.97, as illustrated in Table 1. All coefficients are significant.

**Figure 6:**
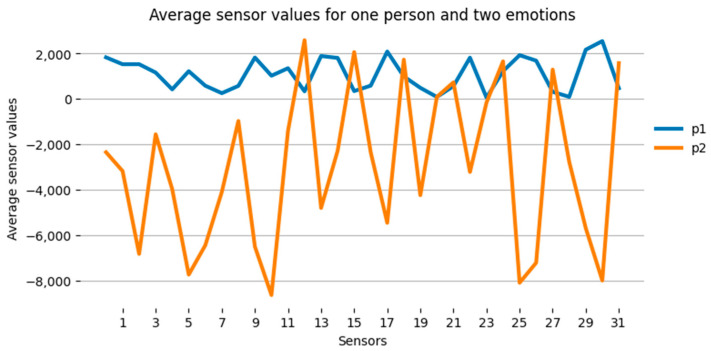
Average values of sensor indications by emotions for the group of subjects: p1—joy, p2—sadness.

Figure 6 shows an example of averaged values of sensor readings for one person when experiencing emotions of joy and sadness.

According to the diagrams presented in Figure 6, we can conclude that, for one person, the sensor values for various emotions differ significantly.

To apply machine learning methods, the digitized sensor signals were normalized, and a matrix of feature objects was created on their basis. The data were divided into training (18 datasets) and test (7 datasets) samples. In order to determine the relationship between the sensor readings that characterize a person’s posture and the emotions experienced, the data were analyzed using well-known machine learning methods. We have used the libraries Scikit-learn and XGBoost in the Python programming language, as well as the tool for automatic selection of parameters for machine learning models GridSearchCV. Table 2 presents a comparison of the training accuracy results for logistic regression (LogisticRegression), k-nearest neighbors (KNeighborsClassifier), reference vector machine (SVM), naive Bayesian classifier (GaussianNB), decision tree (DecisionTreeClassifier), random forest (RandomForestClassifier), and extreme gradient boost (XGBClassifier) methods. Learning accuracy for each model was calculated as the arithmetic mean of the scores for all subjects.

The accuracy on the test sets was calculated using the confusion matrix by the following equation:

$$accuracy=\frac{TP+TN}{TP+FP+FN+TN}$$

where TP is true positive, FP is false positive, TN is true negative, and FN is false negative.

The best result of the classification of the sets from the test sample was obtained using the RandomForestClassifier model. This is the method we chose for further data analysis.

In order to evaluate the possibility of determining emotional state from the data of specific groups of sensors for decision-tree-based machine learning models, the feature_importance method implemented in Python [58] was applied. The value of feature_importance is determined as follows:

(1) $$feature\_importance=\sum_{treesleafs}{\left(v_{1}-avr\right)}^{2}c_{1}+{\left(v_{2}-avr\right)}^{2}c_{2}$$

$$avr=\frac{v_{1}c_{1}+v_{2}c_{2}}{c_{1}+c_{2}}$$

where c_1_ and c_2_ are the total weight of the objects in the left and right leaves, respectively; and v_1_ and v_2_ are the value of Formula (1) for the left and right leaves at the previous tree level.

This parameter allows us to identify the sensors, the triggering of which was most active in certain emotions. The readings of these sensors will be further used to detect emotions.

**Figure 7:**
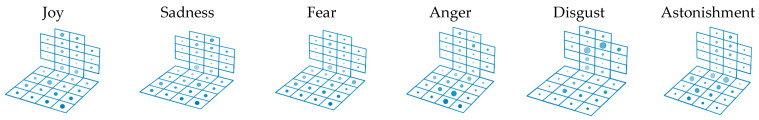
Visualization of the evaluation of the importance of posturometric armchair sensor readings during the formation of certain emotions.

Figure 7 shows the importance of the sensor readings for the group of participants in the experiment. For each emotion, the radius of the dots reflects the importance of the feature.

**Figure 8:**
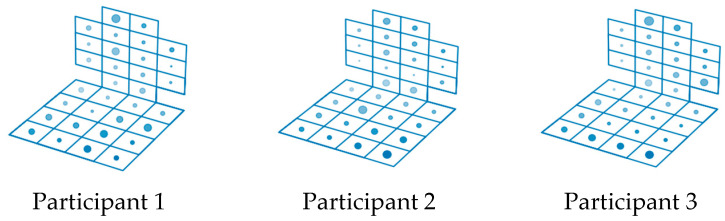
Visualization of posturometric armchair sensor readings during the formation of the joy emotion in three different participants of the experiment.

The analysis of Figure 7 shows that the position of the human body during the various emotions differs, and it is possible to identify similar dependencies of the body position on the experienced emotion for one person. We analyzed the sensor readings for different people during each of the six emotions. Figure 6 illustrates the sensor readings when introducing three different subjects to the emotional state of joy. Figure 8 shows that during the same emotion, the body position of different people is different.

**Figure 9:**
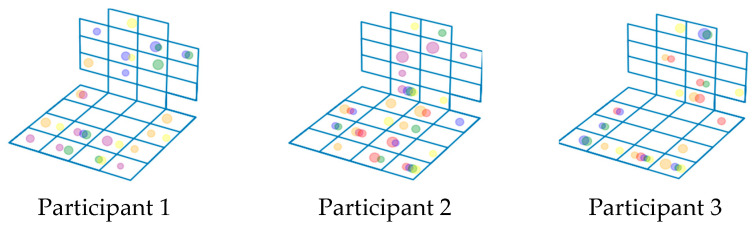
Visualization of posturometric armchair sensor readings during the formation of different emotions in three different participants of the experiment.

Figure 9 shows the distributions of the points characterizing the sensor readings for the different emotions superimposed on a single figure, based on the results of monitoring the postures of the three participants of the experiment. Different colors correspond to the following emotions: joy—yellow, sadness—green, fear—blue, anger—red, disgust—purple, astonishment—orange. You can see that the same sensors are important for identifying different emotions. The important sensors of the chair back are concentrated in the central area for all participants of the experiment; in the seat of the chair for the first and third subjects, the important sensors are located along the perimeter, for the second—in the center.

**Figure 10:**
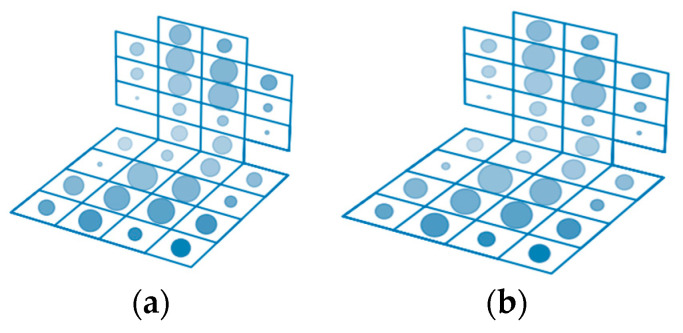
Results of comparing sensor readings for aggregated values for two emotions: (**a**) joy; and (**b**) sadness.

The next step of the study was to compare sensor readings for different emotions for aggregated values for each emotion and for all the test subjects. This means that for all experiment participants, there was a general scheme of sensor operation for the emotion joy, a general scheme for the emotion sadness, etc. Figure 10 shows the result of applying the “arithmetic mean” aggregation function applied to each individual emotion.

As can be seen in Figure 10, for the average values, the sensor readings when a person experiences two different emotions are almost indistinguishable from each other. The results for the other aggregation functions (minimum, maximum, and standard deviation) were similar. The results of the other aggregation functions differ from the results based on the calculation of the arithmetic mean, but are similar to each other.

## 5. Discussion

Comparison of our results with known methods shows significant differences and some advantages of our method.

The posture assessment method using a posturometric armchair is based on posture measurements not requiring sensors to be attached to the human body, in contrast to the assessment method shown in [36], where the inertial measurement unit, together with the control unit, was attached to the ankle, which may be uncomfortable for a person. In addition, during the testing of the system [36], the subjective opinions of the subjects themselves about their condition were used to verify the results. Compared to the work [37], our solution differs in the large number of analyzed pressure points of a sitting person. The advantage of the mentioned work is the consideration of the body mass index. The authors paid more attention to the analysis of horizontal ablations, while our results show the importance of taking into account both horizontal and vertical ablations. In addition, the results of measurements and the corresponding machine learning methods are applied to solve another problem—detection of uncomfortable postures and formation of recommendations on stretching. Pose research using special textile sensors [38] gives good results; however, industrial production is required for such specific sensors. For our system, it is sufficient to use typical strain gauges.

Among the debated issues on the workings of our system is the clarity of the classification of some emotions. Unfortunately, it is difficult to recognize some emotions that are similar in their manifestations. Some researchers, for example, modify the mentioned classifications [46,47,48], reducing the number of emotions. For example, the authors of [59] limited themselves to five emotions (joy, sadness, fear, anger, and disgust), but the same source notes that the main problem in the analysis is the absence of a neutral pose in their classification. In our study, this neutral posture was formed both at the beginning of the tests and between the provoked emotions. The neutral emotion pose provided the calibration of the posturometric armchair.

The limitations of our method can include the Hawthorne effect, which we did not consider in our work. This effect consists of a changing in behavior resulting from awareness of being observed or evaluated [60]; it could appear at the moment of research. However, we believe that the effect was partly compensated for by the Eriksonian hypnosis technique used by the psychologist. Manifestation of this effect when using a chair already calibrated for a particular user for a long time is unlikely, since the person will not be able to control and correct his or her behavior for a long time.

To obtain additional information indirectly indicating some emotional states of the person, we used additional data sources (ECG sensors, GSR, results of video observations, etc.). Information from these sources was used to control the decisions obtained on the posturometric armchair. In the future, these sources can be included in the decision-making process.

In our research, data for creation of a personalized portrait were obtained on the basis of sensory information of the posturometric chair and data obtained during the visual control of the psychologist’s specialist. Using CHI methods will allow us to intellectualize the process of creating a personalized posture portrait: the system will be retrained over a long period of time, which will increase the accuracy of the state assessment.

Studies of the developed models for classifying the state of a person based on the data on his or her body position showed high accuracy. Combining these methods will make it possible to obtain additional results on emotion recognition.

Further research will focus on the creation of multimodal data processing modules, which will use information from additional heterogeneous sources in decision-making in addition to posturometric information. This will allow researchers to form, on the basis of data from sensors of low accuracy, an exact integral indicator; at formation of this indicator, the low accuracy of one type of sensors will be compensated for by readings of sensors with a sensitive element of another nature. It is also considered possible to take into account in the models the biometric indicators of a person (height, weight, BMI), as well as gender differences.

Furthermore, to improve the accuracy of determining the emotional state, the organization of feedback and the formation of a knowledge model that allows one to make decisions about the state of the person, not only based on data, but also on the experience of human–machine interaction with a particular person and taking into account his or her personal characteristics, is needed.

It is planned to develop the work not only to improve the accuracy of human condition assessment, but also to introduce into the system the possibility of its correction by automating existing methods, for example, by regulating breathing.

## 6. Conclusions

This article presents the basis of a system for assessing the emotional state of a person using the parameters of his or her posture in the sitting position. The system is based on a sensory hardware–software system and cognitive architecture combined within the concept of co-evolutionary hybrid intelligence. The sensor system is implemented in the form of a posturometric armchair, which has an original and at the same time simple design that allows us to obtain information about the position of the human body.

On the basis of the sensory information, it is possible to construct a digital model of a person’s body position in various emotional states, which will allow researchers to determine emotions from the data received from the posturometric armchair. At the same time, it is revealed that it is impossible to use one general model for different people. It is necessary to build a personalized portrait of a pose for a concrete person, allowing researchers to identify his or her states.

The system can be used during medical diagnostic procedures and rehabilitation processes. It can also be used in areas where it is necessary to monitor the condition of a person whose professional activity requires concentration to be maintained for a long time, the quick assimilation of new knowledge, good reaction, etc., i.e., in those areas where activity leads to increased psycho-emotional load and can cause cognitive disorders, fatigue, professional burnout, and stress, and eventually can lead to the development of chronic diseases.

## Figures and Tables

**Table 1:** Correlation coefficients of average sensor values for the analyzed emotions.

Emotion	Sadness	Fear	Anger	Disgust	Astonishment
joy	0.944996544	0.82354998	0.97826379	0.94674795	0.90222697
sadness		0.770252641	0.93095542	0.95747521	0.817582839
fear			0.83155515	0.72745024	0.903278
anger				0.97278435	0.932596804
disgust					0.865337527

**Table 2:** Comparison of data processing results on different machine learning models.

			Emotion			
Method	Joy	Sadness	Fear	Anger	Disgust	Astonishment
LogisticRegression	0.9370	0.9692	0.9468	0.9780	0.9782	0.9889
KNeighborsClassifier	0.9451	0.9652	0.9572	0.9658	0.9808	0.9811
SVM	0.9689	0.9760	0.9769	0.9720	0.9848	0.9904
GaussianNB	0.5902	0.7791	0.8059	0.7431	0.8374	0.9443
DecisionTreeClassifier	0.9447	0.9690	0.9608	0.9604	0.9749	0.9805
RandomForestClassifier	0.9721	0.9794	0.9773	0.9745	0.9854	0.9915
XGBClassifier	0.9719	0.9789	0.9772	0.9740	0.9844	0.9908

## Data Availability

Not applicable.
